# Nonconvulsive status epilepticus as an unusual presentation of tuberculous meningoencephalitis: A case report

**DOI:** 10.1002/ccr3.6921

**Published:** 2023-02-10

**Authors:** Seyedehnarges Tabatabaee, Mostafa Almasi‐Dooghaee, Alireza Gandomi‐Mohammadabadi, Seyed‐Mohammad Tabatabaei

**Affiliations:** ^1^ Neurology Department, Firoozgar Hospital, School of Medicine Iran University of Medical Sciences Tehran Iran; ^2^ Student Research Committee, School of Medicine Iran University of Medical Sciences Tehran Iran; ^3^ School of Electrical and Computer Engineering University College of Engineering, University of Tehran Tehran Iran

**Keywords:** electroencephalography, nonconvulsive status epilepticus, seizure, tuberculous meningitis

## Abstract

We describe a 50‐year‐old woman who was recently diagnosed with tuberculosis, with acute alteration in mental status. EEG showed nonconvulsive status epilepticus features. Brain MRI revealed multiple ring‐enhancing lesions. She responded well to treatment for both status epilepticus and tuberculosis. Her level of consciousness improved and she became fully aware.

## Graphical abstract

Particularly in developing countries, Tuberculous meningitis is a very serious condition with a range of clinical presentations. It is important to consider nonconvulsive status epilepticus as one of its various presentation types, since timely intervention can be life‐saving.

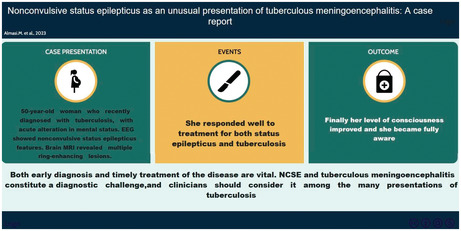

## 1 INTRODUCTION

One of the most serious epileptic conditions is the Nonconvulsive Status Epilepticus (NCSE) which is accompanied by minimal or no motor activity. Electrical activity in NCSE is continuous, lasting at least 30 min and leading to changes in mental or behavioral status.^1^, ^2^ NCSE is relatively common and is responsible for 20%–25% of all reported status epilepticus cases.^3^ It is often difficult to diagnose NCSE, because there is minimal or no objective convulsive activity. Nonetheless underdiagnosis can lead to detrimental results.^4^

Moreover, treatment is not simple and depends on many factors including etiology, electroencephalogram (EEG) findings, and the patient's clinical condition.^3^ NCSE etiologies include idiopathic epilepsy syndrome, metabolic disorders, trauma, brain tumors, cerebral hypoxia, and infectious diseases.^5^

Tuberculous meningoencephalitis (TBM) compromises 1% of all TB cases.^6^ This life‐threatening form of central nervous system (CNS) infection is associated with significantly higher mortality and neurological impairment. TBM has a subacute onset of symptoms with nonspecific clinical signs that may persist for weeks, often making early diagnosis difficult and characterized by fever, headache, vomiting, and focal neurological signs or coma.^7^

Seizures have been reported in 17%–93% of patients with TBM.^8^ The seizures can be either focal or generalized, and convulsive or nonconvulsive.^9^ The etiology of TBM seizures is multifactorial, and has been attributed to meningeal irritation, cerebral edema, tuberculoma, infarction, hydrocephalous, and hyponatremia, either individually or in combination. They can appear either during the active phase or as sequel of meningitis.^10^

Here we report a rare presentation of TBM in a patient who presented with NCSE. This case highlights the importance of clinicians' awareness of unusual clinical presentations of TBM, because NCSE is hard to diagnose unless clinicians are aware of this possibility, as delayed diagnosis is associated with a poor prognosis.

## 2 CASE PRESENTATION

A 50‐year‐old Iranian woman was admitted to our hospital with an exacerbation of abnormal uterine bleeding (AUB) for which surgical treatment was scheduled with total abdominal hysterectomy and bilateral salpingo‐oophorectomy (TAH‐BSO). Her history revealed a 10‐kg weight loss during the previous 3 months, anorexia, night sweats, nausea, vomiting and several episodes of fever and cough, for which she had not sought treatment. Except for primary biliary cirrhosis which diagnosed in 1999, her past medical history was unremarkable. She had been treated with ursodeoxycholic acid (750 mg daily) without immunosuppressive therapy. She had given birth to a son and a daughter. During her admission for TAH‐BSO surgery she had fever. Investigation to find out the underlying cause of fever started. Initial routine blood tests revealed a normal white blood cell count of 8700/mm^3^, low erythrocyte count of 3,630,000/mm^3^, hemoglobin level of 8.3 g/dL, increased C‐reactive protein level of 95 mg/L (normal: <6 mg/L), glucose level of 190 mg/dL, and low sodium level of 130 mmol/L. She had negative results for human immunodeficiency virus. Other laboratory findings showed no remarkable changes. Bilateral pleural effusion was observed in the spiral chest CT scan which lacked lymph node swelling, with diffuse ground‐glass opacities and small centrilobular nodules. Her sputum acid‐fast bacilli test was positive, and antituberculosis treatment was started with isoniazid, rifampicin, ethambutol, and pyrazinamide. As severe uterine bleeding continued, she underwent TAH‐BSO in 3 days. One day after TAH‐BSO surgery, she showed acute alteration of mental status manifested as impaired awareness, disorientation to time, place and person, and bizarre behavior such as undressing herself and urinating in the ward. On the following day she had no awareness to herself and the environment. She was accordingly given neurological and psychiatric consult on the same day because of a suspected psychiatric origin of her symptoms.

Close examination showed negative Kernig's and Brudzinski's signs and mental alterations in the form of impaired awareness and unresponsiveness along with the absence of both meningeal irritation and neck stiffness. The patient was afebrile and normal fundoscopic examination was observed. Eyes showed normal alignment with no deviation and the corneal and pupillary reflexes were also normal. The overall facial expression was symmetric. Plantar reflexes were found to be flexor by eliciting deep tendon reflexes. As the patient did not respond to commands, it was not possible to carry out those neurological tests requiring cooperation, such as cerebellar tests, muscle strength examinations, and sensorium. Nevertheless, the patient moved her limbs symmetrically in bed, with no signs of either convulsion or miniconvulsion.

Cerebrospinal fluid (CSF) was obtained after the neurological examination, about 1 week after her TB treatment was started. Opening pressure was 25 cm H_2_O, leukocyte count was 0 μL, protein level was 36.1 mg/dL, and glucose level was 57 mg/dL (corresponding blood glucose level, 130 mg/dL). Tuberculous DNA polymerase chain reaction (PCR) testing of the CSF showed negative results, but a nested PCR assay yielded a positive result. Adenosine deaminase level was 32.4 IU/L (normal < 10 IU/L).

**FIGURE 1:**
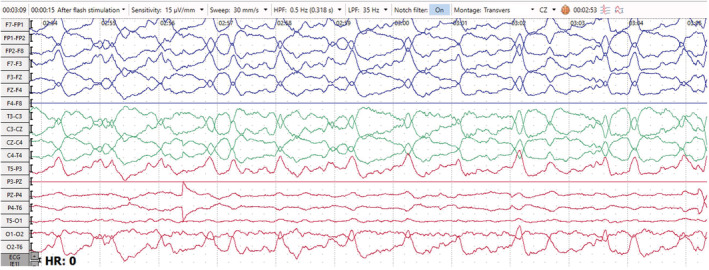
EEG trace in transverse montage (7 μv/mm, 30 mm/s) showing generalized periodic discharges of 1–2 Hz frequency superimposed on a slow background.

**FIGURE 2:**
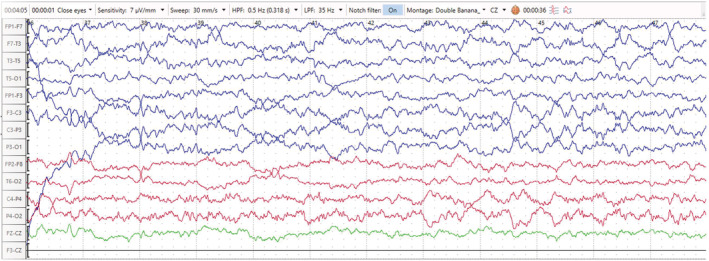
EEG trace in transverse montage (7 μv/mm, 30 mm/s) after the intravenous administration of 10 mg diazepam, showing significant improvement with some transient sharp waves in the left hemisphere and normalization of the background.

**FIGURE 3:**
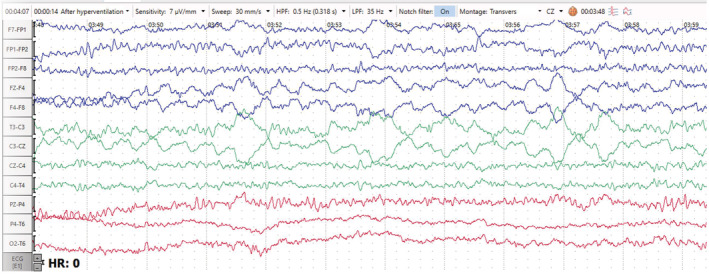
EEG trace in transverse montage (7 μv/mm, 30 mm/s) after antiepileptic treatment, showing significant improvement with a normal alpha background and absence of periodic discharges. Note the C3 and F4 lead artifact is apparent.

The EEG showed generalized 1.5‐ to 2‐Hz continuous sharp and slow wave activities (Figure 1), which improved significantly after the administration of diazepam (Figure 2). After antiepileptic treatment, significant improvement in EEG traces were seen together with a normal alpha background and correction of the periodic discharge waves (Figure 3).

**FIGURE 4:**
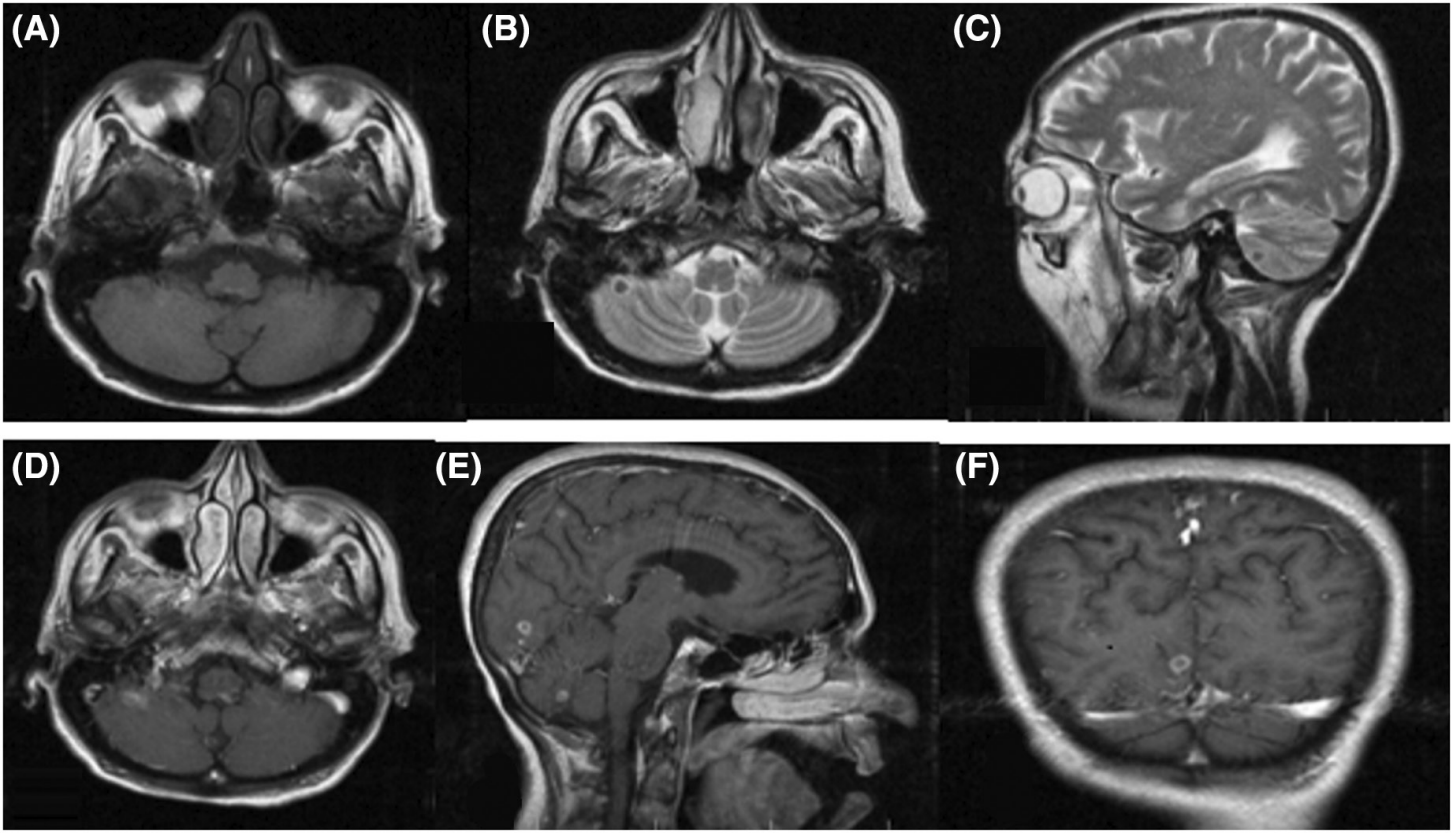
Brain MRI revealed multiple T1‐weighted hyposignal (4‐A) and T2‐weighted hypo/hypersignal (4‐B, 4‐C) lesions with ring‐like gadolinium enhancement in the cerebellum and throughout the cerebellum (4‐D, 4‐E, 4‐F), predominantly at the gray–white matter junction.

Magnetic resonance imaging (MRI) of the brain revealed multiple lesions characterized as iso signal in T1 and low signal at center in T2, with ring enhancement in the supra‐ and infratentorial regions of both hemispheres at the subcortical, white–gray matter junction. Some lesions showed nodular enhancement with moderate vasogenic edema around the lesions. All the findings in the context of the patient's clinical features were consistent with TB (Figure 4). Surgery pathology suggested diffuse granulomatous involvement in favor of TB.

The patient was diagnosed to have TBM presenting with NCSE. Therefore, treatment for NCSE was started. Status epilepticus was first treated by employing a protocol comprising diazepam and levetiracetam infusion, which were followed by a treatment with maintenance oral antiepileptic drugs. Anti‐TBM treatment was resumed with four anti‐tuberculosis medications. The patient's clinical condition and consciousness improved completely after the loading dose of levetiracetam, and she became entirely oriented and aware. No recurrence of cognitive decline was observed in the patient.

## 3 DISCUSSION

This study reports the challenges faced in diagnosing both NCSE and TBM in a patient presented with AUB secondary to uterine tuberculosis. The frequency of status epilepticus in patients with TB is 7.6%.^9^ However, to the best of our knowledge, only four cases of TBM presenting with NCSE have been reported in the literature.^11^, ^12^, ^13^, ^14^

Our patient presented with an altered level of consciousness, unresponsiveness, and persistent seizure activity on the EEG manifested in the form of 1‐ to 2‐Hz continuous sharp and slow wave activity.

Continuous epileptiform EEG activity in NCSE is envisioned to be higher than 2.5 Hz, whereas when epileptiform discharges are less than 2.5 Hz, one out of three other criteria is needed to confirm the diagnosis of NCSE including the presence of miniconvulsive signs, evolution in amplitude or frequency of discharges, and responsiveness to the intravenous administration of benzodiazepines.^15^ Our patient had no miniconvulsive signs but showed an obvious response to intravenous diazepam and levetiracetam, both clinically and in her EEG.

Our patient recovered consciousness and showed normalization of subsequent EEG tracings with antiepileptic therapy. In a study by Kalita et al.^16^ of including 32 patients with highly probable tuberculous meningitis, the main abnormality in their EEG findings was diffuse slowing of the background activity, seen in 69%.

Several reports noted that 84% of CNS tuberculomas clearly showed low signal in T2‐weighted images, and 16% had lesions with central high signal thought to represent caseating necrosis or tuberculous abscesses.^17^, ^18^, ^19^

In the early stage of tuberculoma formation, T1‐ and T2‐weighted images show a mass that is isointense and shows some contrast enhancement, secondary to inflammatory reactions, excess giant cells in the mass, and a poor collagen capsule, which becomes rich in collagen later in the course of disease. The lesions produce low signal in T2‐weighted images because of fibrosis or scar tissue.^20^ Rarely, tuberculomas show calcification on CT, but it can appear as low signal on MRI. Ring‐shaped or nodular contrast enhancement was observed in all tuberculomas.

Almost one third of tuberculomas are multiple.^21^ Various differential diagnoses should be considered in a patient with altered level of consciousness and multiple ring‐enhancing lesions, particularly neoplasms (either metastatic or primary lymphoma) and other infectious diseases such as fungus or bacterial endocarditis. Moreover, tuberculomas have a variety of different features that can mimic other space‐occupying lesions such as neoplasms.

Metastases, multiple gliomas or meningiomas were excluded by further evaluation. No space‐occupying lesions were found in chest or abdominopelvic CT scans. Besides, the pathology findings from her surgery suggested diffuse granulomatous involvement.

Normal CSF glucose and protein levels are unusual but not incompatible with the diagnosis of TB.^22^ Acid‐fast bacilli are seen in only 40% cases on initial CSF examination.^23^ The tuberculin test is negative in 50% of cases at presentation.^24^ As in the study by Arman et al.,^11^ PCR results from the CSF were negative in our patient, although the nested PCR assay was positive. Nested PCR is a technique that reduces nonspecific amplification of the DNA template, and thereby increases the sensitivity and specificity of the reaction, making it useful for suboptimal nucleic acid samples.^25^

The observation of a low signal lesion in T2‐weighted images with nodular or ring‐shaped contrast enhancement in a patient who is coming from a region where tuberculosis is endemic and who suffers from tuberculosis elsewhere in his/her body should alert clinicians to the likelihood of CNS tuberculoma.

Our patient's clinical findings and her unexplained acute change in consciousness, together with chest involvement, diffuse granulomatous infiltration in the uterus and ovaries, and multiple ring enhancement lesions on brain MRI, resulted in the diagnosis of TBM. A further support for this diagnosis comes from the patient's positive response to antituberculosis and antiepileptic treatments.

## 4 CONCLUSION

Various manifestations of CNS tuberculosis, previously a common neurological disorder mainly in developing countries, have now become relevant all over the globe. Both early diagnosis and timely treatment of the disease are vital. NCSE and tuberculous meningoencephalitis constitute a diagnostic challenge, and clinicians should consider it among the many presentations of tuberculosis.

## AUTHOR CONTRIBUTIONS

**SeyedehNarges Tabatabaee:** Data curation; resources; writing – original draft; writing – review and editing. **Mostafa Almasi‐Dooghaee:** Conceptualization; project administration; resources; supervision; validation; writing – review and editing. **Alireza Gandomi‐Mohammadabadi:** Data curation; investigation; writing – original draft; writing – review and editing. **Seyed‐Mohammad Tabatabaei:** Writing – review and editing.

## FUNDING INFORMATION

This research received no specific grant from any funding agency in the public, commercial, or not‐for‐profit sectors.

## CONFLICT OF INTEREST STATEMENT

None.

## CONSENT

Written informed consent was obtained from the patient to publish this report in accordance with the journal's patient consent policy.

## Data Availability

The data that support the findings of this study are available from the corresponding author, upon reasonable request.
